# Pupillary dynamics during hands-off L2 driving and transitions of control under high cognitive load

**DOI:** 10.1371/journal.pone.0355165

**Published:** 2026-08-11

**Authors:** Courtney M. Goodridge, Rafael C. Gonçalves, Ali Arabian, Anthony Horrobin, Albert Solernou, Yee Thung Lee, Audrey Bruneau, Jonny Kuo, Michael G. Lenné, Gaëtan Merlhiot, Yee Mun Lee, Natasha Merat

**Affiliations:** 1 School of Psychology, University of Leeds, Leeds, United Kingdom; 2 Institute for Transport Studies, University of Leeds, Leeds, United Kingdom; 3 Toyota Motor Europe, Brussels, Belgium; 4 Seeing Machines, Canberra, Australia; 5 VEDECOM Institute, Versailles, France; Julius-Maximilians-Universität Würzburg: Julius-Maximilians-Universitat Wurzburg, GERMANY

## Abstract

Arousal plays a vital role in facilitating the human ability to respond flexibly in a goal-directed manner, and pupillometry offers a non-invasive window into arousal-related neural processes given the close relationship between pupil size and Locus Coeruleus-Norepinephrine (LC-NE) activity. Whilst pupillometry has been used to detect cognitive load in manual and automated driving, the dynamic relationship between pre-stimulus (baseline) pupillary state and task-evoked pupillary responses (TEPRs) has not been investigated in driving contexts. This is important because variability in baseline pupil size – itself influenced by cognitive demands such as non-driving related task engagement – may contribute to variability in TEPRs independently of how drivers respond to critical events. This driving simulator experiment aimed to establish whether pupillometry during hands-off Level 2 (L2) driving was a reliable indicator of cognitive load, and to examine whether relationships between pupillary dynamics and behaviour established in controlled laboratory paradigms generalise to applied tasks. The size and reactivity of drivers’ (N = 38) pupils were measured during hands-off L2 driving with and without a cognitive load task, followed by critical and non-critical transitions of control. Analysis revealed that mean, not standard deviation, of pupil diameter was a reliable indicator of cognitive load. Furthermore, higher baseline pupil diameter was associated with smaller TEPRs, and this relationship persisted after correcting for regression to the mean artefacts – suggesting that pre-stimulus pupillary state genuinely constrains subsequent TEPRs. Limited evidence was found that TEPRs or pre-stimulus pupillary variability predicted driver reaction times, potentially reflecting the motoric variability inherent in naturalistic driving responses. Finally, more critical events were associated with larger pupil diameter, indicating that drivers were exerting greater effort to manage the transition. These results indicate that pupillometry is a useful measure of cognitive load and that laboratory-established pupillometric relationships extend, at least partially, to applied driving contexts, with implications for the development of Driver Monitoring Systems (DMS).

## 1. Introduction

### 1.1 Arousal: From theory to application

Arousal is characterised by a combination of psychological and physiological responses to external stimuli (e.g., loud noises) or internal processes (e.g., solving a maths problem) [1], and influences both the available capacity for information processing and the selectivity of attentional allocation towards goal-relevant behaviour [2]. The relationship between arousal and performance is captured by the Yerkes-Dodson law: task performance increases linearly with arousal for simple tasks, but follows an inverted U-shape relationship for difficult tasks, such that too little and too much arousal are detrimental, with optimal performance occurring at intermediate arousal [3]. One proposed mechanism for this inverted- U relationship is that increasing arousal progressively narrows the range of cues an individual attends to; moderate arousal improves performance by filtering out task-irrelevant information; but excessive arousal causes task relevant cues to be excluded, paradoxically narrowing attentional focus whilst increasing distractibility [1,4].

(3), (4), and (2) bring together a shared focus on arousal as the primary governor of attentional focus, with some work extending the arousal system’s role to regulating cognitive control [5,6]. The ubiquitous nature of the arousal system allows it to be applied to real-world tasks. For example, to maintain safe driving performance, a driver should act on the top of this hypothetical performance inverted-U curve [7,8]. However, the monotony of driving in simple conditions for experienced drivers can result in lowered states of awareness, resulting in failures to detect and react to changing road conditions [9,10]. This underload state corresponds to a deviation from the top of the inverted-U curve towards low arousal states and poor task performance. Conversely, overload can occur when physiological arousal is too high, also resulting in detrimental performance. The Cognitive Control Hypothesis (CCH) [11] proposes that driving tasks requiring cognitive control (e.g., negotiating intersections) or those that are non-practiced (e.g., novice drivers in new environments) are more likely to be negatively impacted by increased cognitive load.

Issues associated with underload and overload are likely to be exacerbated as vehicle automation increases; there are concerns that reducing driver mental demands may have detrimental effects on their vigilance and hazard perception [12,13]. Furthermore, drivers are more likely to engage in non-driving related tasks (NDRTs) with increased experience with automated driving functionalities [14–16]. Whilst there is evidence that NDRT engagement can alleviate underload by increasing physiological arousal [17], NDRTs may result in increasing driver cognitive load beyond optimal levels [18,19]. These issues are particularly problematic for intermediate levels of automation (e.g., SAE Level 2 [L2] systems) where the driver is required to monitor the road environment and intervene when necessary.

Despite the implicit assumption that cognitive load negatively impacts transitions of control, research evidence does not always corroborate this. Steering and braking reaction times to obstacle avoidance events have consistently been unaffected by increased cognitive load [18,20–22]. This might be because reactions to strong looming signals (e.g., a decelerating lead vehicle) do not rely upon cognitive control; rather, they are automatised responses that are triggered by bottom-up saliency of visual looming – and thus are not negatively impacted [11,18]. The quality of transitions of control *have* been found to degrade with elevated cognitive load in some instances; the time between steering initiation and lane crossing increased with cognitive load [20]. Lane changes are responses that are likely to involve cognitive control [23]; drivers must engage top-down attentional control to shift their gaze between the wing mirrors, the road centre, an obstacle they want to overtake, and the destination lane [24,25]. Findings from [20] may, therefore, point towards evidence of a transition of control subtask that is detrimentally impacted by cognitive load.

The CCH suggests that manual driving responses that are novel or inherently uncertain are more likely to rely on cognitive control and, consequently, be negatively impacted under high cognitive load. Given that transitions of control are relatively infrequent, they are likely to remain less practiced than routine driving behaviours and may place greater demands on cognitive control processes. If these assumptions hold, fluctuations in arousal may directly influence the cognitive mechanisms required for successful transitions, highlighting the importance of understanding arousal’s role in applied driving contexts. Hence the concept of Driver Readiness (DR) – defined as the probability of a successful takeover [26] – becomes central. Although efforts have been made to standardise DR metrics [27–30], it remains an abstract construct that is not directly observable and is therefore inferred from related measures. Cognitive load has emerged as a key predictor of successful transitions of control, positioning it as a critical component of DR. If the arousal system fundamentally governs cognitive performance, it is essential that future Driver Monitoring Systems (DMS) can reliably capture physiological indices of arousal. Pupillometry, given its close links to underlying neural dynamics, represents a promising candidate for real-time assessment in this domain.

### 1.2 Linking arousal to pupillary and neural dynamics

Aside from the pupil light response (PLR), pupil size varies as a function of a variety of cognitive phenomena including mental effort [31–33], decision making [34,35], surprise [36,37], and neural gain [38,39]. These Task-Evoked Pupillary Responses (TEPRs) are typically involuntary pupil dilations that peak any time from 500 ms [40] to 2 s post stimulus [41]. TEPRs follow systematic changes in neural activity throughout the cortex, regulated via neuromodulators [42]. Therefore, the pupillary response has been proposed as an easily measurable physiological reflection of arousal and attention networks [1,43].

Cognitively driven modulations of pupil size intersect through three main nuclei: (1) the Pretectal Olivary Nucleus (PON), which receives top-down information from brain areas implicated in higher order visual-saccadic processing [44,45]; (2) the Superior Colliculus (SC), which integrates sensory signals to facilitate the orientation of cross-modal attention [46]; and (3) the Locus Coeruleus (LC), which receives input from a variety of brain regions and is a primary source of norepinephrine (NE) (see [47] for a review). The LC-NE system is of particular interest because it has been hypothesised to regulate cortical arousal [48], alongside interacting with top-down cortical systems associated with attentional control and executive function [49,50]. The LC-NE system is therefore heavily involved in task-related shifts of arousal [51,52].

LC-NE-centred neural circuits have been heavily associated with pupillary responses [47,53]. Animal studies have revealed that pupillary dynamics in response to acoustic or effort-related stimuli positively correlate with LC activity [54,55]. Similarly, stimulation of the LC in monkeys and rats produced pupil dilations [54,56]. In humans, blood oxygenation level dependent (BOLD) signals in the LC have been positively correlated with pupil size when completing cognitive tasks [35,51]. Whilst pupil size is not a direct readout of LC activity, their close relationship provides a means for measuring changes in attentional state mediated by LC-NE activity [43,51].

### 1.3 Pupillary dynamics and applied task performance

Pupillometry offers a non-invasive window into brain processes associated with arousal and attention given the relationship between LC activity and pupillary responses. It is no wonder then, that researchers within the automotive domain have focused on pupillometry to understand cognitive resource engagement. In manual driving, conversational NDRTs [57], encountering unexpected pedestrians [58], and overtaking truck platoons [59] increase pupil size, indicating an increase in cognitive load during these interactions. During SAE Level 2 and Level 3, larger pupil size was also associated with smaller time headways and transitions of control due to increased cognitive demand [60].

However, pupillometry in driving contexts predominantly focus on tonic (e.g., sustained) pupil diameter as an index of cognitive load. Yet, sustained pupil diameter leaves largely unexamined whether the dynamic relationship between pre-stimulus (baseline) pupillary state and TEPRs is preserved in applied driving contexts. Research in controlled laboratory paradigms has established that pre-stimulus pupil size predicts the magnitude of subsequent TEPRs [61–63]. These studies propose that this relationship reflects the behaviour of task-evoked (phasic) LC responses that depend upon baseline (tonic) LC activity. Phasic responses to task-relevant events are thought to be maximised at intermediate tonic levels but attenuated when tonic activity is either too low or too high [53]. Adaptive Gain Theory (AGT) summarises this relationship between pupil size, LC activity, and goal directed behaviour. However, it is hard extrapolating pupillary data to LC-NE dynamics given the indirect relationship. Furthermore, an overlooked phenomenon is that large pre-stimulus pupils are likely to decrease in size afterwards, simply because larger pupils a more likely to become smaller [41]; thus requiring corrections of these regression to the mean artifacts [64].

It is important to outline that the baseline-TEPR relationships, and investigations of regression to the mean artefacts, have not been studied in driving. This is a key omission, because baseline pupil size varies considerably across individuals and situations in driving – including as a direct consequence of NDRT engagement – meaning that variability in pre-stimulus state will contribute to variability in TEPRs independently of how participants respond to critical events. Without understanding this relationship, it is not possible to determine whether a smaller TEPR in response to a request to intervene (RTI) reflects reduced responsiveness to the event itself or is simply a consequence of an elevated pre-stimulus baseline. Examining baseline-TEPR relationships in the context of transitions of control therefore has implications for the practical development of DMS that may use TEPRs to index driver state.

TEPRs have also been proposed as indicators of the degree to which attentional and cognitive resources are mobilised in response to a specific event [63]. In controlled laboratory paradigms, larger TEPRs have been associated with faster reaction times, suggesting that the magnitude of TEPRs reflect the efficiency of cognitive engagement with an incoming stimulus [65,66]. If this relationship generalises to driving, TEPRs in response to an RTI could provide an index of how effectively a driver is engaging with a transition of control. Examining whether TEPR magnitude predicts reaction time during transitions of control therefore represents an important test of whether laboratory-established pupillometric relationships extend to applied settings, with direct implications for whether TEPRs could serve as a useful real-time index of driver responsiveness in DMS.

Finally, a further dimension of pupillary dynamics that has received recent attention is short-term pupillary variability, indexed by the Micro-Pupillary Unrest Index (M-PUI). Rather than capturing the average level or evoked change in pupil size, M-PUI quantifies moment-to-moment fluctuations in pupil diameter over short time windows and has been proposed as an index of transient vigilance state [67]. In psychomotor vigilance tasks, higher pre-stimulus M-PUI has been associated with slower reaction times, suggesting that periods of greater pupillary instability reflect momentary lapses in alertness [68]. This is particularly relevant in the context of automated driving, where sustained monitoring of a system that rarely requires intervention may produce fluctuating vigilance states [12,69]. If M-PUI captures these transient fluctuations in vigilance, it may be sensitive to variability in DR that precedes a transition of control. However, this has not been tested in driving contexts, and it remains unclear whether the relationship between M-PUI and reaction time observed in simple laboratory tasks survives the increased complexity and response variability inherent in transitions of control.

### 1.4 Current work

Safe and effective transitions of control depend on the driver’s readiness to intervene, which is strongly influenced by underlying arousal and cognitive control processes. However, DR cannot be measured directly and must be inferred from physiological and behavioural indicators. Pupillometry offers a promising, non-invasive index of these processes, yet it remains unclear whether theoretically grounded predictions of pupillary dynamics generalise to complex, real-world driving contexts.

The analysis in this manuscript is structured into three parts. First, we evaluate pupillometric indices as objective measures of cognitive load during hands-off L2 driving, focusing on the mean and standard deviation (SD) of pupil diameter as candidate metrics, given their relevance as indicators of arousal-related processing. In the second part, we examine three aspects of pupillary dynamics motivated by the broader pupillometry literature [65,66,68]. First, we investigate whether larger baseline pupil diameter is associated with smaller TEPRs during transitions of control, and whether this relationship persists after correcting for regression to the mean artefacts [64]. Second, we examine whether TEPR magnitude predicts driver reaction time during transitions of control. Third, we investigate whether pre-stimulus pupillary variability, indexed by M-PUI, is associated with driver reaction time. In the third part, we examine how task demands influence sustained pupil dynamics during transitions of control by manipulating cognitive load (N-back) and event criticality. Prior work has shown that mean pupil diameter increases with mental effort in both controlled tasks [70,71] and applied driving contexts [58–60,72]. We extend this by testing whether more critical driving events elicit greater sustained increases in pupil diameter, reflecting heightened arousal and task engagement during transition scenarios.

## 2. Method

### 2.1 Participants

41 participants originally took part in the experiment. However, three participants were removed because they failed to follow the experimental instructions or because eye tracking data was not captured correctly. The remaining 38 participants had a reasonable gender split between males (N = 22) and females (N = 16), an average age of 38.81 years (range = 22–65 years old, SD = 12.502), all had normal or corrected to normal vision, had a valid UK driving license for an average of 17.84 years (range = 4–43 years, SD = 12.119), and were regular drivers who drove an average of 9355.26 miles annually (range = 5000–20000 miles, SD = 4153.975).

### 2.2 Apparatus and materials

The experiment was conducted at the University of Leeds Driving Simulator, consisting of a Jaguar S-type cab encased within a 4 m spherical with a 300° field of view projection dome. The virtual environment created for this driving simulator experiment was kept at daytime luminance levels throughout. Therefore, the impact that changes in environmental lighting could have on pupil reactivity was experimentally controlled from the offset. Longitudinal and lateral movement was provided via a hexapod motion base and a 5 m x 5 m X-Y table. Pupillometry data were captured using the Seeing Machines’ Driving Monitoring System (DMS) eye tracker, sampling at 60 Hz. The DMS – comprised of a driver-facing infrared camera – utilised proprietary algorithms to derive head position, facial feature detection, eyelid position, and pupil and gaze tracking to monitor the drivers’ visual attention. The system monitored driver’s gaze direction and identified gaze targets towards the forward roadway or towards other regions. The NASA Task Load Index (NASA-TLX) was used to measure subjective workload [73].

### 2.3 Design and procedure

Informed consent was obtained, both written (in terms of signing a consent form) and verbally (by asking the participant if they consented to take part in the research), and standardised instructions were delivered. All procedures were approved by the University of Leeds Research Ethics Committee (Reference code: 2022-0353-206). Participants were recruited for this study between 27/09/2024–28/10/2024. The original design of the experiment was 2 x 2 Repeated Measures design. The two within-participant factors were event criticality (i.e., the time to collision [TTC] at the onset of a lead vehicle braking) and the manipulation of driver cognitive load. The event criticality was manipulated over two levels: TTC = 3 s corresponded to severe scenarios and TTC = 5 s corresponded to less severe scenarios [18,74–76]. Driver cognitive load was also manipulated over two levels; a no-load condition and a high load condition where participants completed an auditory version of the verbal response delayed digit recall task (N-back, where N = 2) (see [77] for details) during hands-off L2 driving. Digits were presented via the vehicle speaker system, whilst participant responses were verbal. Participants had the opportunity to practice the N-back task at the start of the experiment without any driving. Analysis of N-back performance and subjective workload ratings can be found in previous work [18,78].

Before the main experimental procedure, participants conducted a practice run to familiarise themselves with the simulator dynamics and the driving system. Participants were taught how to engage the L2 system and where to place their hands and feet when the L2 system was on. The experimental procedure consisted of two main drives – one where drivers had to complete N-back whilst monitoring their L2 system, and another where they just had to monitor the road and the driving system. Drive order was counterbalanced across the sample, and each drive lasted approximately 35 minutes. Each drive comprised of 10 discrete events. Following each drive, the NASA-TLX was used to measure subjective workload. Each event consisted of 30 s of manual driving, followed by approximately two minutes of hands-off L2 driving. After two minutes of hands-off L2 driving, a request to intervene (RTI) was delivered by a short auditory tone, and the simultaneous change in colour of a steering wheel icon positioned in the dash area from steady green (automation engaged) to flashing red (intervention required) (see [18,78] for more details on the procedure). Four of these events were critical, which involved a lead vehicle braking sharply, creating a rear-end scenario that drivers had to avoid. The remaining six were non-critical, which involved scenarios where there was no lead vehicle, or the lead vehicle did not brake. These were included to guard against learning effects such that the presence of a lead vehicle was not always associated with a critical event. Critical and non-critical events were presented in a pseudo-random order. Transitions of control could be initiated via 3 different modes: turning the steering wheel in either direction by more than 2°, pressing any of the operational pedals, or pressing a micro-switch button strapped to the steering wheel. For non-critical trials, a majority of transitions (77%) were initiated with the microswitch; for critical trials, a majority of transitions (84%) were initiated by using the operational pedals. Participants were instructed to respond as quickly and as safely as possible. Overall reaction time calculated as the fastest time between the RTI until the L2 system had been deactivated.

### 2.4 Data preparation and statistical modelling

#### 2.4.1 Pupillometry pre-processing.

Eye blinks were automatically identified and tagged via the Seeing Machines’ Driving Monitoring System. To account for pupil size drop-offs, samples before and after blinks were also removed. The pupil time course was linearly interpolated across blinks. Mean pupil size was then computed by averaging the left and right pupil diameter. For investigating tonic pupil diameter during hands-off L2 driving and during the transition of control, and TEPRs in response to RTI, the pupil time series was bandpass filtered (third order butterworth, passband = 0.05–4 Hz). For the calculation of M-PUI, a Hanning filter was used to remove high frequency fluctuations. In line with previous research [68], we investigated four different time windows for the Hanning filter (50 ms, 100 ms, 200 ms, and 400 ms) to assess whether correlations between M-PUI and reaction times were consistent across filtering parameters.

For investigating pupil diameter during hands-off L2 driving, we calculated the mean and SD of pupil diameter during the 2-minute driving period. For investigating increases in pupil diameter associated with the transition of control, we calculated mean pupil diameter during the transition of control window. For calculating TEPRs, two time-based epochs were used. The first was the pre-RTI baseline epoch (−500-0 ms) and the second was the stimulus response epoch (300–1800 ms). For each trial, we subtracted these two epochs to give our primary dependent measure for assessing phasic pupillary dilations: change in pupil response. The lower bound of the stimulus response epoch was chosen to eradicate implausibly fast pupillary dilations; the fastest a pupil reacts is within 200 ms [41]. The upper bound stimulus response epoch was chosen because – although pupillary dilations associated with orienting attention often peak 500 ms post-stimulus [40,79] – if there is a clearly defined triggering stimulus that requires the engagement of arousal, pupillary dilation can peak up to 1000 ms post-stimulus [41,80].

A key prediction from laboratory paradigms is that higher baseline pupil diameter should be associated with smaller TEPRs [41,61,81]. However, a correlation like this can also come about through regression to the mean given that pupils that are already large are more likely to constrict than become larger due to physiological constraints rather than cognitive related phenomena [41,64]. One way of correcting for this regression to the mean artefact is to analyse the spontaneous tendency of the pupil to change size on the time scale of an RTI-evoked pupil dilation, as a function of baseline pupil size. To implement this correction, we used a method proposed by [64]. We estimated the spontaneous tendency of the pupil to change as a function of baseline diameter using a pseudo-baseline and a pseudo-change window that was not time-locked to the transitions of control (i.e., a period where drivers were monitoring the L2 system). A cubic regression model was then fitted to predict the pseudo-change from baseline pupil size. This model was then used to predict the expected baseline-dependent change in pupil size for each trial based on its pre-stimulus baseline. The predicted change was subtracted from the observed TEPR, yielding a corrected measure of phasic pupil response. If higher baseline pupil diameter was still associated with smaller TEPRs following this baseline correction, this would suggest that the pre-stimulus pupillary state genuinely constrained the magnitude of subsequent TEPRs, rather than the correlation being a statistical consequence of regression to the mean.

Finally, we calculated M-PUI to estimate short-term vigilance levels. First, we applied a Hanning filter defining four different time windows prior to the RTI (50 ms, 100 ms, 200 ms, and 400 ms). For each of the smoothed time series datasets, M-PUI was calculated as the absolute value for the degree of change in pupillary fluctuations divided by the length of the pupillary time series.

#### 2.4.2 Reaction time pre-processing.

For investigating the relationship between reaction times and pupillary dynamics during transitions of control, implausibly small reaction times were filtered out of the dataset. We set a threshold of 300 ms based on previous research investigating human reactions to collision avoidance warning systems [82] (28 in total). Next, we calculated z scores and used these to remove long reaction times that did not represent valid behaviour; we set a threshold of 3 SDs above the mean. Overall, all reaction times in the final dataset were between 0.3–4.2 s.

#### 2.4.3 Statistical modelling.

A Bayesian approach was used to model the data. For each analysis, a Bayesian multilevel model was fitted. The distribution of each dependent variable was assessed prior to the analysis, and the appropriate distribution family was chosen for model building. Posterior distributions were estimated using the No-U-Turn sampler in the brms package in R [83] and weakly informative regularising priors were used. Weakly regularising priors attempt to “regularise” the posterior distribution by keeping it within reasonable bounds without trying to influence the data as much as possible. This helps rule out implausible effects; for example, predicting increases in pupil diameter that are physiologically implausible. Data, analysis code, and models can be found in the following link: https://osf.io/vmxj3.

Separate design matrices were specified for the three parts of the analysis, reflecting the different task demands associated with these phases. However, for all models, we used treatment coding for the categorical variables inputted into the model. Analysis of tonic pupil diameter during hands-off L2 driving focused on sustained cognitive demands preceding the RTI. The mean and SD of pupil diameter were calculated for each hands-off L2 driving segment and modelled as a function of an intercept ($\beta_{\text{0}}$), N-back ($\beta_{N}$), lead vehicle ($\beta_{L}$) and an interaction term between these variables ($\beta_{N:L}$). Both critical and non-critical trials were included; however, event criticality was not modelled, as no overt response to a developing hazard had occurred during the period where pupil size was measured.

TEPRs were modelled as a function of an intercept ($\beta_{\text{0}}$), baseline pupil diameter ($\beta_{B}$), N-back ($\beta_{N}$), event criticality ($\beta_{{TTC}_{\text{3}}}$, $\beta_{{TTC}_{\text{5}}}$), and all two way ($\beta_{B:N}$*,* $\beta_{B:{TTC}_{\text{3}}}$*,* $\beta_{B:{TTC}_{\text{5}}}$*,* $\beta_{N:{TTC}_{\text{3}}}$*,* $\beta_{N:{TTC}_{\text{5}}}$)*,* and three way interactions ($\beta_{B:N:{TTC}_{\text{3}}}$*,* $\beta_{B:N:{TTC}_{\text{5}}}$) between the predictors. The inclusion of these higher-level interactions was because we hypothesied that baseline pupil diameter may have interacted with the presence of N-back and event criticality when impacting TEPRs. Models were fitted before and after correction for regression to the mean effects to assess whether effects qualitatively changed following corrections. Reaction time was modelled as a function of TEPRs ($\beta_{TEPR}$), event criticality ($\beta_{TTC}$), two way interactions between TEPRs and event criticality ($\beta_{TEPR:{TTC}_{\text{3}}}$, $\beta_{TEPR:{TTC}_{\text{5}}}$), a two way interaction between TEPR and N-back ($\beta_{TEPR:N}$), and three way interactions between N-back, event criticality, and TEPRs ($\beta_{TEPR:{TTC}_{\text{3}}:N}$, $\beta_{TEPR:{TTC}_{\text{5}}:N}$). Main effects of N-back and the two way interaction between N-back and event criticality were omitted given that previous research analysed these data and found no effect on reaction times [18]. Reaction time was also modelled using the same parameters except the M-PUI metric replaced the TEPR metric. For the analysis of increases in pupil diameter during the transition of control, mean pupil diameter was modelled as a function of an intercept ($\beta_{\text{0}}$), N-back ($\beta_{N}$), event criticality ($\beta_{{TTC}_{\text{3}}}$, $\beta_{{TTC}_{\text{5}}}$), and two-way interactions between these predictors ($\beta_{N:{TTC}_{\text{3}}}$, $\beta_{N:{TTC}_{\text{5}}}$).

Each parameter was associated with probability distributions quantifying the level of uncertainty, conditioned on the data. Posterior distributions of parameters were described by their mean and a 95% Credible Interval (CI) [84]. The *probability of direction* (pd) was also reported for each fixed effect parameter, defined as the probability that an effect was positive or negative [85].

## 3. Results

### 3.1 Effect of N-back and lead vehicle presence on pupil diameter during hands-off L2 driving

**Fig 1:**
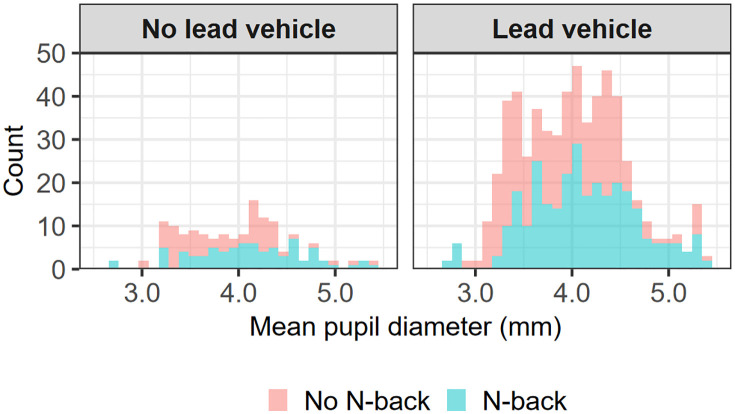
Distributions of mean pupil diameter during hands-off L2 driving with and without N-back and lead vehicle presence.

First, we assessed the distributional properties of mean pupil diameter during hands-off L2 driving across N-back and lead vehicle conditions (see Fig 1).

**Fig 2:**
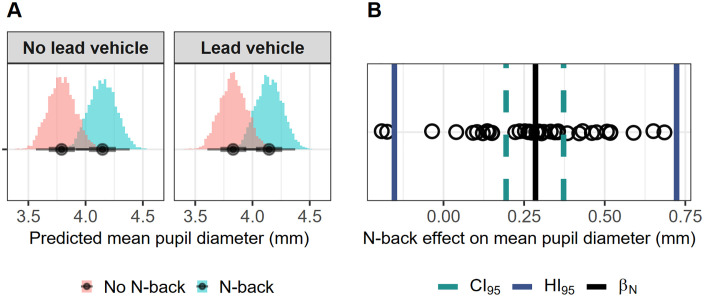
A) Posterior predictions of expected mean pupil diameter as a function of lead vehicle and N-back. Mean pupil diameter is predicted to increase during N-back relative to no N-back conditions B) Strip plot displaying a range of effects of N-back on mean pupil diameter. The black line denotes the average increase in mean pupil diameter, (fixed effect), the green dashed lines denote the heterogeneity of the average effect of N-back (95% Credible Intervals) and the blue solid lines denote the population heterogeneity of the effect of N-back.

A Bayesian model specifying a Gaussian distribution was fitted to assess changes in mean pupil diameter as a function of N-back and lead vehicle. The model (see Table 1) predicted a small increase in mean pupil diameter (0.032, [CI: −0.006, 0.050]) when lead vehicles were present (3.865 mm) relative to when they were not present (3.833 mm) during hands-off L2 driving. Despite this, the 95% credible intervals suggested that this effect could be close to 0. Conversely, the model predicted a strong effect of N-back. Mean pupil diameter increased (0.285, [CI: 0.192, 0.369]) when drivers completed the N-back task (4.118 mm) relative to the no N-back drives (3.833 mm). Further investigation of the heterogeneity of this average effect revealed that 90% of the population would be expected to demonstrate similar increases in pupil diameter as a function of cognitive load during hands-off L2 driving. This is reflected in the current sample; only three participants demonstrated small *reductions* in pupil diameter whilst completing N-back (see Fig 2B).

**Table 1:** Posterior means and 95% CIs for parameters predicting mean pupil diameter.

	Coefficient (95% CI)	pd
$\beta_{0}$	**3.833 (3.650, 4.022)**	**100%**
$\beta_{N}$	**0.285 (0.194, 0.372)**	**100%**
$\beta_{L}$	**0.032 (−0.006, 0.070)**	**95.02%**
$\beta_{NL}$	−0.035 (−0.088, 0.020)	89.05%
Participants	38	
Observations	744	

**Fig 3:**
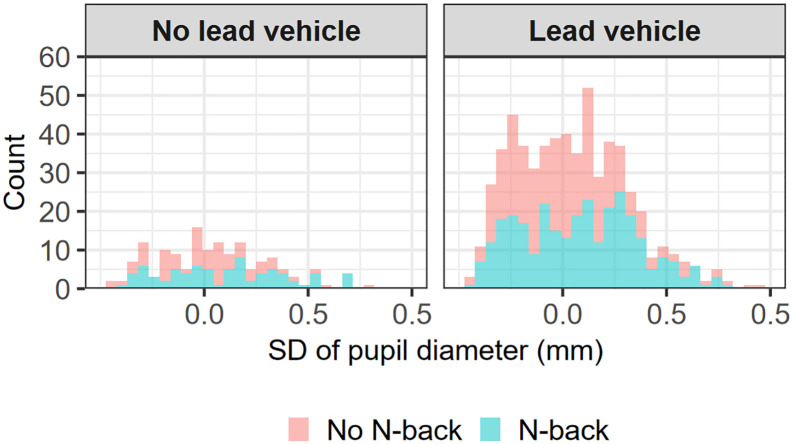
Distributions of the SD of pupil diameter during hands-off L2 driving with and without N-back and lead vehicle presence.

Next, we assessed the distributional properties of the SD of pupil diameter across N-back and lead vehicle conditions (see Fig 3).

**Fig 4:**
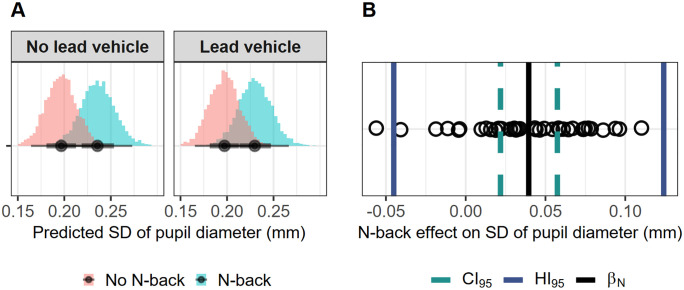
A) Posterior predictions of expected SD of pupil diameter as a function of lead vehicle and N-back. SD of pupil diameter is predicted to increase during N-back relative to no N-back conditions. However, this increase appears smaller and less certain than for mean pupil diameter B) Strip plot displaying a range of effects of N-back on SD of pupil diameter. The black line denotes the average increase in SD of pupil diameter, (fixed effect), the green dashed lines denote the heterogeneity of the average effect of N-back (95% Credible Intervals) and the blue solid lines denote the population heterogeneity of the effect of N-back.

Modelling revealed no effect of lead vehicle; however, N-back was associated with increased SD of pupil diameter (0.039, [CI: 0.021, 0.057]), (0.236 mm) relative to no N-back conditions (0.196 mm) (see Table 2 and Fig 4). Further investigation of the heterogeneity of the average effect revealed that only 82% of the population would be predicted to have similar increases in the SD of pupil diameter as a function of N-back; almost a fifth of drivers demonstrated no differences or reversals of the average effect. Whilst these findings support previous work indicating that pupillometry can be a useful indicator of increased cognitive load [60], the current analysis reveals that mean, rather than the SD of pupil diameter, is likely to be a more reliable measure.

**Table 2:** Posterior means and 95% CIs for parameters predicting SD of pupil diameter.

	Coefficient (95% CI)	pd
$\beta_{0}$	**0.196 (0.163, 0.229)**	**100%**
$\beta_{N}$	**0.039 (0.021, 0.057)**	**100%**
$\beta_{L}$	0.001 (−0.008, 0.009)	55.03%
$\beta_{NL}$	−0.006 (−0.019, 0.006)	84.38%
Participants	38	
Observations	744	

### 3.2 Correlations between baseline pupil diameter and TEPRs

**Fig 5:**
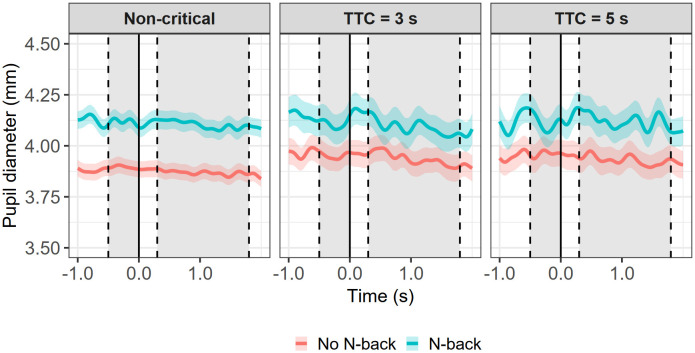
Mean pupil responses during N-back (in blue) and no N-back (in pink) conditions. The coloured shaded regions represent the standard error of the mean. Pre-stimulus baseline (−500-0) and stimulus response epochs (300-1800) are shaded in grey.

Next, we examined the relationship between baseline pupil diameter and TEPRs. Mean pupil response was plotted for each condition, with the pre-stimulus baseline and stimulus response epochs shaded in grey (see Fig 5).

**Fig 6:**
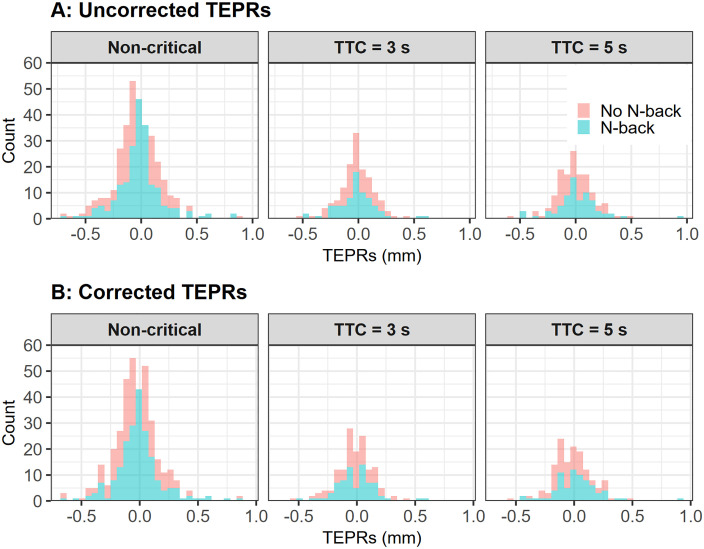
Distributions of corrected and uncorrected TEPRs within each event criticality and N-back condition.

The distribution of uncorrected (see Fig 6A) and corrected (see Fig 6B) TEPRs were plotted to assess distributional properties; a Gaussian model was then used for fitting.

**Fig 7:**
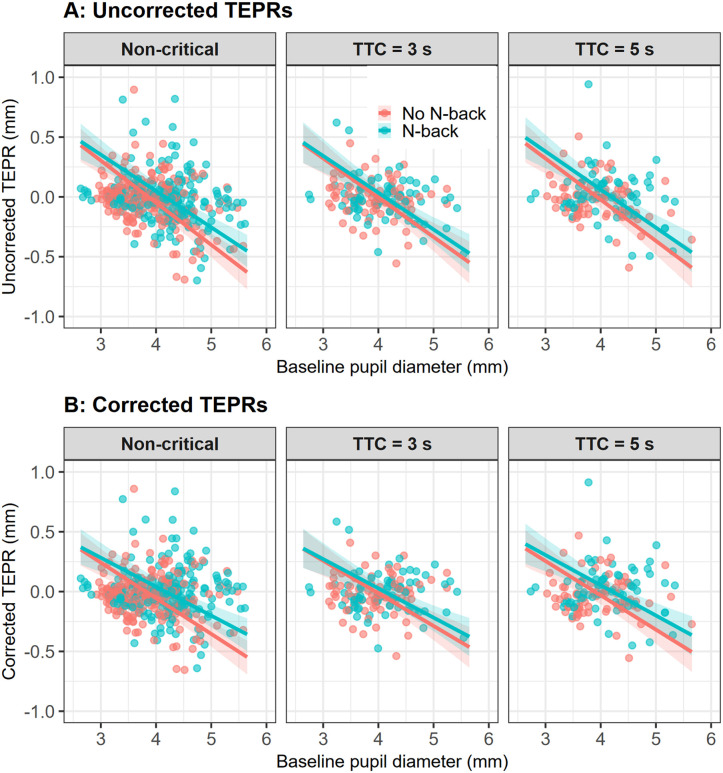
A negative correlation between baseline pupil diameter and uncorrected (A) and corrected (B) TEPRs. This indicates that pre-stimulus pupillary state genuinely constrains the magnitude of subsequent TEPRs, rather than the correlation being a statistical consequence of regression to the mean.

The Bayesian model using the uncorrected TEPR revealed no effect of N-back or event criticality on TEPRs. However, there was evidence that higher baseline pupil diameter was associated with smaller TEPRs (−0.325 mm, [CI: −0.409, −0.249]) (see Fig 7A). Furthermore, the model revealed an interaction between baseline pupil diameter and N-back; the negative correlation between baseline pupil diameter and TEPR was stronger following no N-back trials (−0.341 mm, [CI: −0.427, −0.263]) relative to N-back trials (−0.309 mm, [CI: −0.394, −0.224]). These effects were maintained even after correcting for regression to the mean artefacts; whilst the relationship between baseline pupil diameter and TEPRs was smaller (−0.266 mm, [CI: −0.346, −0.191]) (see Fig 7B; Table 3), the interaction effect between no N-back (−0.285 mm, [CI: −0.367, −0.209]) and N-back trials (−0.246 mm, [CI: −0.336, −0.158]) remained similar in size.

**Table 3:** Posterior means and 95% CIs for parameters predicting TEPRs.

Parameters	Uncorrected TEPR model		Corrected TEPR model	
	Coefficient (95% CI)	pd	Coefficient (95% CI)	pd
$\beta_{0}$	**1.359 (1.005, 1.005)**	**100%**	**1.136 (0.796, 1.470)**	**100%**
$\beta_{B}$	**−0.352 (−0.433, −0.266)**	**100%**	**−0.298 (−0.376, −0.216)**	**100%**
$\beta_{N}$	−0.095 (−0.307, 0.111)	81.13%	−0.128 (−0.337, 0.085)	88.90%
$\beta_{{TTC}_{\text{3}}}$	−0.050 (−0.336, 0.234)	63.57%	−0.060 (−0.337, 0.213)	66.83%
$\beta_{{TTC}_{\text{5}}}$	−0.005 (−0.270, 0.267)	51.83%	−0.021 (−0.290, 0.258)	57.32%
$\beta_{B:N}$	**0.048 (−0.002, 0.100)**	**96.98%**	**0.056 (0.004, 0.107)**	**98.25%**
$\beta_{B:{TTC}_{\text{3}}}$	0.023 (−0.048, 0.095)	73.42%	0.025 (−0.043, 0.095)	76.02%
$\beta_{B:{TTC}_{\text{5}}}$	0.007 (−0.061, 0.075)	58.93%	0.011 (−0.058, 0.079)	64.07%
$\beta_{N:{TTC}_{\text{3}}}$	0.051 (−0.324, 0.425)	60.62%	0.058 (−0.310, 0.427)	62.25%
$\beta_{N:{TTC}_{\text{5}}}$	0.076 (−0.306, 0.450)	65.33%	0.076 (−0.298, 0.449)	65.65%
$\beta_{B:N:{TTC}_{\text{3}}}$	−0.026 (−0.119, 0.066)	71.03%	−0.028 (−0.119, 0.062)	72.38%
$\beta_{B:N:{TTC}_{\text{5}}}$	−0.022 (−0.115, 0.072)	68.82%	−0.022 (−0.116, 0.070)	68.50%
Participants	38		38	
Observations	706		706	

### 3.3 Correlations between TEPRs and reaction time

**Fig 8:**
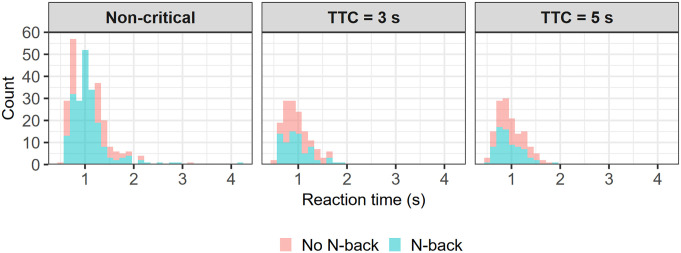
Distributions of raw reaction times with and without N-back for each event criticality.

Next, we analysed the relationship between driver reaction times and TEPRs. Distributions of reaction time data demonstrated characteristic negative skew (see Fig 8). As such, a log normal distribution was used to account for the extended right tail.

**Fig 9:**
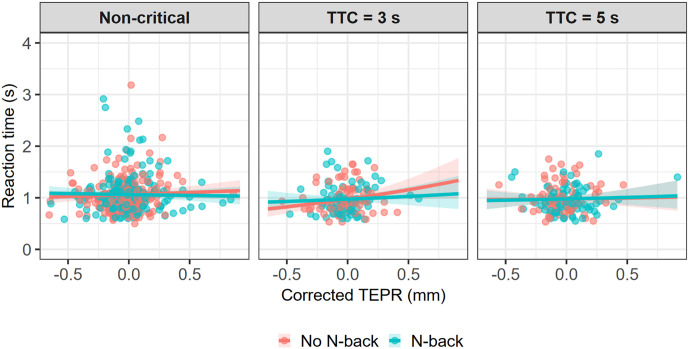
Relationship between TEPRs and reaction time within each N-back and event criticality condition.

Modelling revealed no effect of corrected TEPR on reaction times (see Fig 9). However, we did find that for typical TEPRs, reaction times were faster for event criticalities of 3 s (−0.093 s, [CI: −0.137, −0.049]) and 5 s (−0.083 s, [CI: −0.129, −0.040]) relative to non-critical transitions (see Table 4).

**Table 4:** Posterior means and 95% CIs for parameters predicting reaction time based on TEPRs.

	Coefficient (95% CI)	pd
$\beta_{\text{0}}$	0.032 (−0.023, 0.089)	88.63%
$\beta_{TEPR}$	0.073 (−0.090, 0.241)	80.35%
$\beta_{{TTC}_{3}}$	**−0.087 (−0.132, −0.042)**	**100%**
$\beta_{{TTC}_{5}}$	**−0.083 (−0.128, −0.038)**	**100%**
$\beta_{TEPR:N}$	−0.106 (−0.329, 0.115)	82.80%
$\beta_{TEPR:{TTC}_{\text{3}}}$	0.263 (−0.074, 0.602)	93.08%
$\beta_{TEPR:{TTC}_{\text{5}}}$	−0.043 (−0.379, 0.283)	59.60%
$\beta_{TEPR:{TTC}_{\text{3}}:N}$	−0.129 (−0.588, 0.318)	70.50%
$\beta_{TEPR:{TTC}_{\text{5}}:N}$	0.132 (−0.299, 0.558)	73.87%
Participants	38	
Observations	706	

### 3.4 Correlations between M-PUI and reaction time

**Fig 10:**
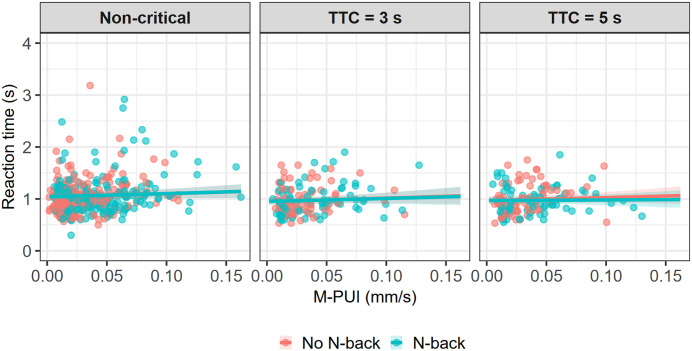
Relationship between M-PUI (50 ms time window) and reaction time within each N-back and event criticality condition.

Next, we analysed the relationship between driver reaction times and M-PUI. We fitted a separate model for each filter time window. Overall, we found limited evidence for the relationship between reaction time and M-PUI (see Table 5, see Fig 10). For small time windows (50 ms), there was some evidence that higher M-PUI was associated with longer reaction times. However, the pd indicated only a 94% probability that this correlation would be above zero. As time windows increased, the size and reliability of this effect reduced, as highlighted by the reduced size of the coefficient and pd, respectively.

**Table 5:** Posterior means and 95% CIs for parameters predicting reaction time based on M-PUI.

Parameters	50 ms time window		100 ms time window		200 ms time window		400 ms time window	
	Coefficient (95% CI)	pd	Coefficient (95% CI)	pd	Coefficient (95% CI)	pd	Coefficient (95% CI)	pd
$\beta_{\text{0}}$	0.010 (−0.049, 0.071)	63.42%	0.013 (−0.045, 0.072)	67.30%	0.018 (−0.039, 0.080)	71.97%	0.022 (−0.036, 0.079)	77.65%
$\beta_{M-PUI}$	0.569 (−0.161, 1.298)	94.05%	0.531 (−0.265, 1.306)	91.12%	0.483 (−0.313, 1.293)	87.87%	0.344 (−0.533, 1.226)	77.20%
$\beta_{{TTC}_{3}}$	**−0.087** **(−0.142, −0.034)**	**99.97%**	**−0.088** **(−0.140, −0.037)**	**99.98%**	**−0.087** **(−0.136, −0.036)**	**100.00%**	**−0.086** **(−0.134, −0.040)**	**100.00%**
$\beta_{{TTC}_{5}}$	**−0.069** **(−0.126, −0.013)**	**99.27%**	**−0.071** **(−0.124, −0.018)**	**99.57%**	**−0.074** **(−0.124, −0.024)**	**99.75%**	**−0.077** **(−0.125, −0.029)**	**99.88%**
$\beta_{M-PUI:N}$	0.007 (−0.594, 0.604)	51.23%	0.034 (−0.631, 0.701)	53.82%	0.055 (−0.685, 0.791)	56.17%	0.049 (−0.789, 0.881)	55.02%
$\beta_{M-PUI:{TTC}_{\text{3}}}$	0.065 (−0.746, 0.872)	56.12%	0.075 (−0.794, 0.949)	57.10%	0.031 (−0.868, 0.926	52.80%	0.011 (−0.941, 0.934)	51.03%
$\beta_{M-PUI:{TTC}_{\text{5}}}$	−0.023 (−0.892, 0.852)	52.90%	−0.023 (−0.894, 0.859)	52.03%	−0.049 (−0.936, 0.842)	53.87%	−0.012 (−0.979, 0.920)	50.97%
$\beta_{M-PUI:{TTC}_{\text{3}}:N}$	−0.078 (−0.892, 0.735)	57.20%	−0.043 (−0.911, 0.796)	53.62%	−0.048 (−0.950, 0.819)	53.73%	−0.038 (−1.000, 0.905)	52.33%
$\beta_{M-PUI:{TTC}_{\text{5}}:N}$	−0.467 (−1.297, 0.346)	87.15%	−0.443 (−1.295, 0.423)	84.42%	−0.367 (−1.269, 0.512)	79.10%	−0.203 (−1.133, 0.738)	66.32%

### 3.5 Increases in pupil diameter during transitions of control

**Fig 11:**
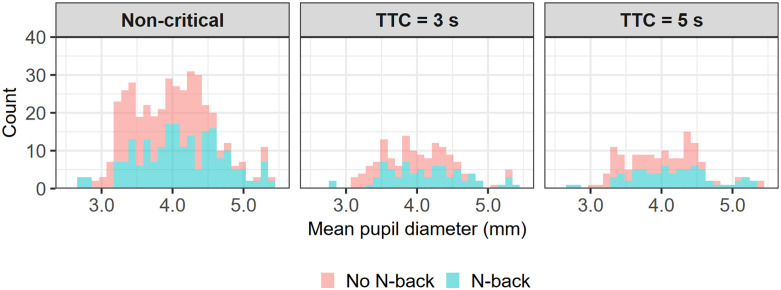
Distributions of mean pupil diameter during transitions of control with and without N-back for each event criticality.

Finally, we investigated tonic increases in pupil diameter associated with general increases in effort during the transitions of control. The transition of control time window was based on previous research [60]; 10 s from the RTI plus the average reaction time (~ 1 s). Mean pupil diameter during this window was used to investigate whether more critical events were associated with increased effort during the transition of control. Distributions of mean pupil diameter were plotted (see Fig 11) and as such, a Gaussian model was selected.

**Fig 12:**
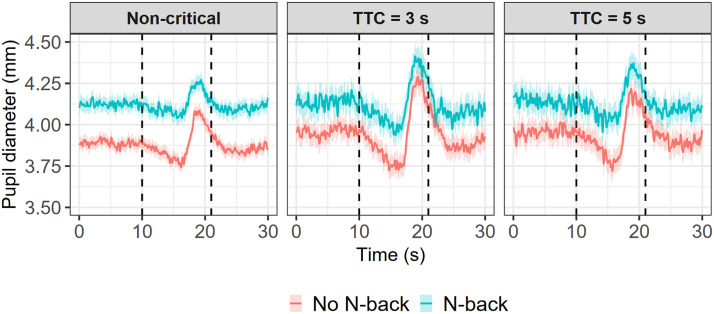
Average pupil diameter over time pre-RTI (0-10 s), during the transitions of control (10-21 s), and post transition of control (21-30 s).

Fig 12 shows average pupil diameter divided into three sections: pre-RTI (0–10 s), during the transition of control (10–21 s), and post transition of control (21–30 s). For each condition, it was evident that there were large tonic increases in pupil diameter several seconds after the RTI (e.g., the first dashed vertical line in each panel of Fig 12) and after the initial disengagement of the automated system (approximately 1 s after the RTI).

Modelling mean pupil diameter during the transition of control time window revealed an interaction effect between N-back and event criticality conditions (see Table 6). For no N-back conditions, average pupil diameters for TTC = 3 s conditions (3.923 mm) were larger than for non-critical conditions (3.857 mm) (0.065, [CI: 0.022, 0.103]); a similar effect was observed between TTC = 5 s conditions (3.932 mm) and non-critical conditions (0.075, [CI: 0.037, 0.113]). Conversely, the model revealed no consistent changes between the event criticalities during N-back conditions: mean pupil diameters in TTC = 3 s conditions (4.140 mm) were not reliably larger than non-critical conditions (4.141 mm) (0.001, [CI: −0.045, 0.039]); nor were TTC = 5 s conditions (4.146 mm) reliably larger than non-critical conditions (0.005, [CI: −0.031, 0.044]). Overall, these results are in-line with the hypothesis that more critical events are likely to increase physiological arousal as indicated by increases in pupil diameter. However, they also point towards ceiling effects in the N-back condition such that altering the criticality of an event did not further increase pupil diameter (and thus our measure of tonic arousal).

**Table 6:** Posterior means and 95% CIs for parameters predicting tonic pupil diameter increases.

	Coefficient (95% CI)	pd
$\beta_{0}$	**3.857 (3.675, 4.036)**	**100%**
$\beta_{N}$	**0.282 (0.201, 0.366)**	**100%**
$\beta_{{TTC}_{3}}$	**0.065 (0.024, 0.105)**	**99.90%**
$\beta_{{TTC}_{5}}$	**0.075 (0.035, 0.112)**	**99.98%**
$\beta_{N:{TTC}_{3}}$	**−0.065 (−0.118, −0.014)**	**99.85%**
$\beta_{N:{TTC}_{5}}$	**−0.069 (−0.121, −0.017)**	**99.28%**
Participants	38	
Observations	707	

### 3.6 Post-hoc analysis: driver gaze during transitions of control

**Fig 13:**
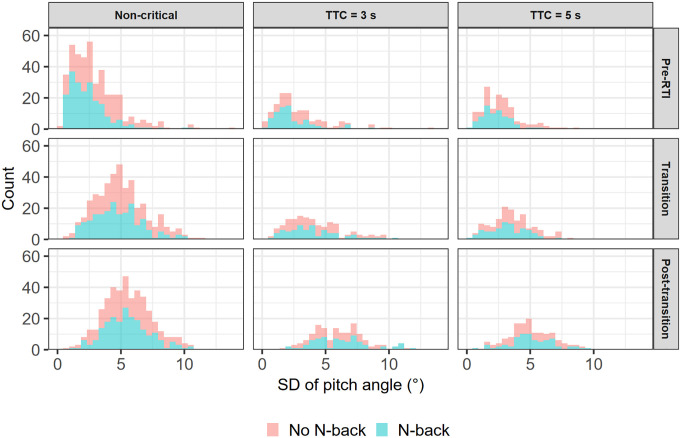
Distributions of the SD of pitch angle each time window with and without N-back for each event criticality.

Finally, we conducted a post-hoc analysis of driver gaze distribution during transitions of control to understand where drivers were sampling visual information from. We calculated the SD of pitch angle during each of the three-time windows, and within each N-back and event criticality condition. Based on distributions of the raw data (see Fig 13), a Gaussian Bayesian model was fitted to investigate the effects. Once again, we used treatment coding for all categorical variables in the model. Thus we modelled SD of pitch angle as a function of an intercept ($\beta_{\text{0}}$), N-back ($\beta_{N}$), event criticality ($\beta_{{TTC}_{\text{3}}}$, $\beta_{{TTC}_{\text{5}}}$), time window ($\beta_{{TW}_{transition}}$, $\beta_{{TW}_{post-transition}}$), all two way interactions ($\beta_{N:transition}$*,* $\beta_{N:post-transntion}$*,* $\beta_{{TTC}_{\text{3}}:transition}$*,* $\beta_{{TTC}_{\text{5}}:transition}$*,* $\beta_{{TTC}_{\text{3}}:post-transition}$*,* $\beta_{{TTC}_{\text{5}}:post-transition}$ $\beta_{N:{TTC}_{\text{3}}}$*,* $\beta_{N:{TTC}_{\text{5}}}$), and three way interactions ($\beta_{N:{TTC}_{\text{3}}:transition}$*,* $\beta_{N:{TTC}_{\text{5}}:transition}$*,* $\beta_{N:{TTC}_{\text{3}}:post-transition}$, $\beta_{N:{TTC}_{\text{5}}:post-transition}$).

**Fig 14:**
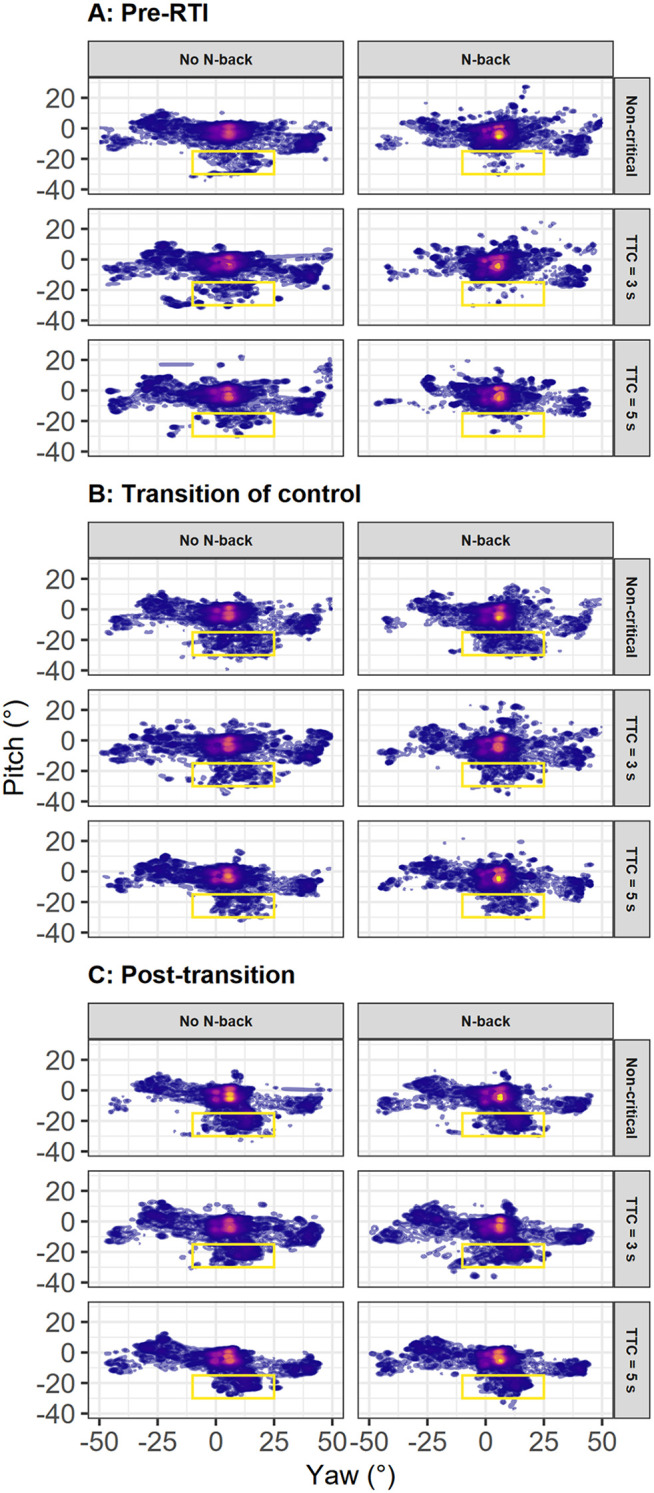
Contour plots show the average distribution of gaze during A) the 10 s prior to the RTI, B) the transition of control, and C) the 10 s following the transition of control. The yellow box highlights approximate location of the dashboard area. Drivers appeared to gaze more towards the dash area during the transition and post-transition phase relative to the pre-RTI phase.

Modelling revealed two prominent interaction effects (see Table 7). Firstly, during the pre-RTI time window, SD of pitch angle was consistent for non-critical (3.397°), TTC = 3 s (3.333°), and TTC = 5 s (3.123°) conditions. However during the transition of control, SD of pitch angle increased to varying extents; the increase was largest for non-critical conditions (5.2289°) (2.191, [CI: 1.717, 2.656]) relative to TTC = 3 s conditions (4.258°) (0.927, [CI: 0.387, 1.542]) and TTC = 5 s conditions 3.854° (0.731, [CI: 0.107, 1.296]). Overall, this was indicative of drivers focusing more on the forward roadway during critical transitions control; conversely, drivers seemed to be looking down more during non-critical transitions, presumably looking for and obtaining vehicle status information (see Fig 14).

**Table 7:** Posterior means and 95% CIs for parameters predicting SD of pitch angle.

Parameters	Coefficient (95% CI)	pd
$\beta_{0}$	**3.397 (3.027, 3.755)**	**100.00%**
$\beta_{N}$	**−1.005 (−1.306, −0.713)**	**100.00%**
$\beta_{{TTC}_{\text{3}}}$	−0.066 (−0.480, 0.347)	62.08%
$\beta_{{TTC}_{\text{5}}}$	−0.274 (−0.700, 0.152)	89.98%
$\beta_{{TW}_{transition}}$	**1.831 (1.413, 2.257)**	**100.00%**
$\beta_{{TW}_{post-transition}}$	**2.191 (1.723, 2.663)**	**100.00%**
$\beta_{N:{TTC}_{\text{3}}}$	0.075 (−0.510, 0.664)	60.23%
$\beta_{N:{TTC}_{\text{5}}}$	0.189 (−0.423, 0.781)	73.85%
$\beta_{N:transition}$	**0.469 (0.044, 0.896)**	**98.58%**
$\beta_{N:post-transition}$	**0.899 (0.493, 1.336)**	**100.00%**
$\beta_{{TTC}_{3}:transition}$	**−0.904 (−1.476, −0.321)**	**99.88%**
$\beta_{{TTC}_{5}:transition}$	**−1.099 (−1.697, −0.508)**	**100.00%**
$\beta_{{TTC}_{\text{3}}:post-transition}$	0.370 (−0.211, 0.967)	89.68%
$\beta_{{TTC}_{\text{5}}:post-transition}$	−0.274 (−0.862, 0.325)	81.78%
$\beta_{{N:TTC}_{\text{3}}:transition}$	0.235 (−0.563, 1.079)	70.78%
$\beta_{N:{TTC}_{\text{5}}:transition}$	−0.297 (−1.132, 0.527)	75.15%
$\beta_{N:{TTC}_{\text{3}}:post-transition}$	0.347 (−0.460, 1.173)	80.30%
$\beta_{N:{TTC}_{\text{5}}:post-transition}$	−0.045 (−0.885, 0.813)	54.70%
Participants	38	
Observations	2118	

The second interaction effect was between N-back and time window. We found that during the pre-RTI phase, SD of pitch was lower during N-back conditions relative to no N-back conditions (−1.005, [CI: −1.303, −0.711]). This is consistent with evidence indicating that drivers were visually tunneling during N-back trials. However, during the transition of control (−0.535, [CI: −0.837, −0.226]), and post-transition (−0.105, [CI: −0.398, 0.198]) time windows, the difference between N-back and no N-back conditions were much smaller, once again indicating that drivers were looking down more during these phases regardless of their level of cognitive load.

## 4. Discussion

This investigation examined pupillary dynamics during hands-off L2 driving and transitions of control. Modelling revealed that mean pupil diameter was a more reliable indicator of cognitive load than the SD of pupil diameter; mean pupil diameter increased during N-back trials, with 90% of the population predicted to show similar increases, compared to only 82% for the SD. We also examined three relationships between pupillary dynamics and behaviour established in controlled laboratory paradigms. A robust negative relationship between baseline pupil diameter and TEPRs was found, and crucially, this persisted after correcting for regression to the mean artefacts – suggesting that pre-stimulus pupillary state genuinely constrains subsequent evoked responsiveness rather than the correlation being a statistical consequence of physiological boundaries. This pattern is consistent with theoretical accounts linking tonic arousal state to the magnitude of stimulus-evoked neural responses, including accounts that implicate LC-NE dynamics in regulating the balance between sustained and evoked pupillary activity [35,54,86,87]. However, we found limited evidence that TEPRs or pre-stimulus pupillary variability predicted driver reaction times, likely reflecting the motoric variability introduced by naturalistic driving responses. Finally, more critical transitions of control were associated with larger increases in pupil diameter, consistent with heightened arousal and effortful task re-engagement following the RTI. A post-hoc analysis additionally revealed that drivers directed their gaze more towards the forward roadway during critical transitions, whereas non-critical transitions were associated with more time spent looking towards the dashboard area, presumably to obtain vehicle status information – a pattern consistent with prior research on driver gaze behaviour during transitions of control [76,88,89].

### 4.1 Pupillometry during hands-off L2 driving

Assessing whether an average effect is consistent across a population is important given that understanding variations in driver cognitive load is vital if DMS are to reliably and accurately detect driver state. For example, according to the European New Car Assessment Programme (Euro NCAP) 2026, as a minimum requirement, Forward Collision Warning (FCW) and/or Automated Emergency Braking (AEB) systems should differ by a least 200 ms between a distracted/impaired driver and an attentive driver in order to account for longer driver reaction times. The analysis reported here suggests that the SD of pupil diameter was not a reliable indicator of impairment in this context; and that despite an average increase, almost a fifth of the population would be expected to demonstrate null or opposite effects under conditions of high load. This is problematic because an algorithm detecting positive changes in the SD of pupil diameter may incorrectly conclude that no changes in cognitive load are present during periods of high cognitive load (i.e., false negatives).

Overall, these findings suggest that mean pupil diameter is a more robust candidate than short-term variability measures for monitoring changes associated with increased cognitive demand during hands-off L2 driving. Importantly, this should not be interpreted as indicating that larger pupils necessarily reflect higher absolute arousal in all circumstances, as pupil diameter is influenced by multiple physiological and environmental factors [90]. Rather, under controlled luminance conditions, the results demonstrate that mean pupil diameter provides a more consistent marker of changes associated with increased cognitive demands than the SD of pupil diameter. For practical DMS applications, this suggests that algorithms should prioritise sustained changes in pupil diameter, while combining pupillometry with environmental sensing to account for non-cognitive influences on pupil size [91,92].

### 4.2 TEPRs in an applied context

Larger pre-stimulus pupil diameter was negatively associated with subsequent TEPRs, and this association survived correction for regression to the mean artefacts. This finding is consistent with AGT – a unifying account linking arousal, pupil size, and behavioural performance via LC-NE dynamics [53]. AGT proposes that the strength of task-evoked (phasic) LC responses depends on baseline (tonic) LC activity. When tonic LC activity is low, task-related events elicit relatively weak phasic responses, corresponding to reduced engagement and poorer performance typically observed in low arousal states. At intermediate tonic levels, phasic LC responses are maximised, enhancing neural gain and supporting optimal task performance. In contrast, elevated tonic LC activity attenuates phasic responses while promoting more indiscriminate increases in neural gain, resulting in reduced selectivity and greater distractibility. Controlled laboratory studies have found similar correlations between pre-stimulus (tonic) pupil size and TEPRs [61,63,81]. We found similar effects in a more applied task setting, a pattern *consistent with* the tonic-phasic trade-off predicted by AGT, though the present data cannot confirm LC-NE involvement directly.

Previous research had also reported negative correlations between TEPRs and response times [53,61,63,65,93], suggesting that larger TEPRs were associated with faster responses. This relationship is thought to reflect NE release from LC projections, increasing the gain of neural interactions and facilitating more efficient information processing [53,93], which would be expected to reduce response latency. However, the present study found no relationship between TEPRs and reaction time. One possible explanation is the presence of multiple potential takeover actions. These responses (e.g., braking, steering, or pressing a button) differ in their minimum motor execution latencies, as reflected in the slightly slower reaction times observed for non-critical transitions of control (which were predominantly initiated via a button press rather than operational pedals). Such variability in motor demands may have obscured any underlying relationship between pupillary dynamics and reaction time. In contrast, controlled laboratory studies typically require participants to respond in highly controlled and uniform ways. Although allowing drivers to respond naturally increases ecological validity, it also introduces motoric variability that may reduce sensitivity to detect associations between TEPRs and behavioural responses.

Evidence for an association between M-PUI and reaction times was limited. A small relationship was observed when M-PUI was computed over short time windows, but this effect attenuated as window length increased. This pattern is broadly consistent with prior findings linking elevated M-PUI to slower responses in psychomotor vigilance tasks [68] and suggests that M-PUI may be most sensitive to transient fluctuations in vigilance. One interpretation is that shorter windows better capture moment-to-moment variability in arousal-related processes, whereas longer windows may average over these fluctuations, reducing sensitivity to their behavioural consequences. Similarly to the relationships between reaction times and TEPRs, the relatively weak effects observed in the present driving context may also reflect increased variability in behavioural responses. Unlike psychomotor vigilance tasks, which typically involve simple button presses, driving responses involve more complex and variable motor actions, which may reduce the detectability of subtle vigilance-related influences on reaction time.

More broadly, the present findings demonstrate that relationships between baseline pupil diameter and subsequent TEPRs reported in tightly controlled laboratory paradigms also emerge during transitions of control in automated driving. However, these data should not be interpreted as identifying specific arousal states or confirming the operation of LC-NE mechanisms. Rather, they indicate that pre-stimulus pupillary state constrains subsequent pupillary responsiveness in a predictable manner. For DMS development, this suggests that interpreting TEPRs without accounting for baseline pupil diameter may underestimate or overestimate responsiveness to RTIs. Incorporating baseline correction into TEPR-based algorithms may therefore improve the interpretation of task-evoked pupillary responses.

### 4.3 Pupillometry during transitions of control

Consistent with prior work demonstrating increased pupil diameter during transitions of control [60], we found that more critical transitions – manipulated via TTC – were associated with larger increases in pupil diameter. Interestingly, this effect was observed only in the no N-back condition. One possible explanation for this is a ceiling effect: the N-back manipulation increased overall pupil size, potentially limiting further dilation during critical transitions due to physiological constraints. Crucially, the present design does not allow us to determine whether increases associated with event criticality reflect elevated cognitive load specifically, or more general physiological arousal associated with critical events. Previous research suggests that responses to critical rear-end scenarios may be highly automatised [18], implying that the earliest behavioural reactions are unlikely to require substantial cognitive control. In this context, increased pupil diameter could plausibly reflect sympathetic activation – a “fight-or-flight” response triggered by imminent collision threat. Supporting this interpretation, pupil diameter has been shown to predict accident severity in real-world contexts [94], suggesting sensitivity to acute threat. Notably, however, the pupil increases observed in the present study emerged several seconds after the transition of control. This delayed elevation is less consistent with a purely reflexive sympathetic response and may instead reflect the sustained effort required to re-engage with the dynamic driving task after monitoring a hands-off L2 system. From this perspective, critical transitions may demand increased cognitive control, error monitoring, and vigilance, resulting in sustained arousal adjustments rather than brief phasic responses.

Taken together, these findings suggest that sustained pupil diameter during the transition itself may provide a useful physiological indicator of the overall demands imposed by a takeover event. However, these increases should not be interpreted as direct measures of arousal, but rather as reflecting the combined cognitive and behavioural demands associated with managing the transition. For practical applications, this suggests that DMS could benefit from monitoring changes in tonic pupil diameter across the takeover period, rather than relying solely on pre-transition measurements, to identify situations in which drivers are experiencing elevated task demands.

### 4.4 Future work and conclusions

Although there is a close relationship between pupil diameter and LC activity, the mechanism is not direct and involves many interconnected pathways [47]. Future research may want to confirm this relationship through functional neuroimaging techniques such as electroencephalography (EEG) or functional Near-Infrared Spectroscopy (fNIRs). Whilst fNIRs does not have the spatial resolution to image the LC, the LC does extensively innervate the cerebral cortex, including the dorsolateral and dorsomedial prefrontal cortex (PFC), which fNIRs has access to [53,95–97]. Hence the combined use of fNIRs and pupillometry could elucidate the interactions between the LC and the PFC in applied tasks. The P300 event related potential (ERP) has been proposed as an electrophysiological reflection of the phasic activity of the LC-NE system [98]. There is evidence to suggest that pupil diameter and the P300 amplitude are sensitive to similar experimental parameters [99,100]. Large P300 amplitudes are associated with large TEPRs during oddball tasks [101,102]. This is particularly interesting given that P300 amplitudes are seen to be *dampened* during auditory oddballs presented during states of high cognitive load in manual and automated driving [103–106]. Taken in context with the current results, there appears to be circumstantial evidence that phasic pupillary responses and the P300 component could be associated in a driving context; albeit more robust evidence would be needed to confirm this. This association may have safety related implications given that high cognitive load has also been found to reduce P300 amplitudes towards driving related sensory information (e.g., lead vehicle brake light onset) [103]. If TEPRs demonstrate similar behaviour, this could be a useful non-invasive indication as to whether a driver has attended to, and encoded, task-relevant information in the road environment during non-critical and critical situations.

Future work may also want to further investigate the impact that drowsiness has on pupillometry. The Pupillary Unrest Index (PUI) is a common measure of sleepiness that uses pupillometry, however it requires a standardised experimental procedure for calculation [107]. It might be interesting to understand whether M-PUI – which is calculated over much smaller timescales – is associated with drowsiness. This could be operationalised in a number of ways. For example, studies may want to investigate whether M-PUI is impacted by the length of time participants are using an automated system given that extended use may result in reduced alertness [108,109]. Similarly, the relationship between M-PUI and reliable subjective (the Karolinska Sleepiness Scale [KSS]) and objective (percentage of eye closure [PERCLOS], and amplitude velocity ratio [AVR]) measures of alertness could be assessed [110–113].

In conclusion, this study demonstrates that pupillometry can provide a sensitive physiological measure of changes associated with cognitive demands during hands-off L2 driving and transitions of control. Mean pupil diameter reliably distinguished periods of increased cognitive load, while baseline pupil diameter consistently constrained the magnitude of subsequent TEPRs, even after accounting for regression to the mean artefacts. Importantly, these findings should not be interpreted as direct evidence of specific arousal states or LC-NE activity. Rather, they show that well-established relationships between different pupillary measures observed in controlled laboratory paradigms also generalise, at least in part, to a complex applied driving task. From a practical perspective, the results suggest that DMS could benefit from combining sustained pupil diameter with baseline-adjusted TEPRs when interpreting driver state, while integrating these measures with other behavioural and environmental information. More broadly, this work helps define what pupillometry can – and cannot – tell us about DR, providing a more principled foundation for the development of physiologically informed monitoring systems for road vehicles.
